# Draft Whole-Genome Sequence of a *Haemophilus quentini* Strain Isolated from an Infant in the United Kingdom

**DOI:** 10.1128/genomeA.01075-16

**Published:** 2016-10-06

**Authors:** Alasdair T. M. Hubbard, Sian E. W. Davies, Laura Baxter, Sarah Thompson, Mark M. Collery, Daniel C. Hand, Colin G. Fink, D. John I. Thomas

**Affiliations:** a Micropathology Ltd., University of Warwick Science Park, Coventry, United Kingdom; b School of Life Sciences, University of Warwick, Coventry, United Kingdom; c Microbiology Department, Sheffield Teaching Hospitals NHS Foundation Trust, Northern General Hospital, Sheffield, United Kingdom

## Abstract

*Haemophilus quentini* is a rare and distinct genospecies of *Haemophilus* that has been suggested as a cause of neonatal bacteremia and urinary tract infections in men. We present the draft whole-genome sequence of *H. quentini* MP1 isolated from an infant in the United Kingdom, aiding future identification and detection of this pathogen.

## GENOME ANNOUNCEMENT

A distinct genospecies of nontypeable *Haemophilus* that is clinically relevant has recently been described which falls into the group *Haemophilus influenzae* biotype IV, with the name “*Haemophilus quentini*” having been proposed for the group (1–6). *Haemophilus quentini* has been found in the male urogenital tract and has been suggested as the cause of urinary tract infections in men, albeit very rarely (1, 3). *Haemophilus quentini* has also been recently identified as the cause of bacteremia in neonates in Italy and China (2, 4), in both cases it was isolated and identified through 16s rRNA gene sequencing (2, 4).

An unknown culture was isolated from an infant in the United Kingdom and was identified to be *Haemophilus haemolyticus* through sequencing of the outer membrane protein following amplification with primers targeting the P6 outer membrane protein of *Haemophilus* species and comparing the sequence to those available in public databases. However, it was also identified as *H. quentini* through sequencing of the 16s rRNA gene, similar to how it was previously identified in Italy and China (2, 4). The lack of homology of the P6 outer membrane gene product was therefore found to be due to there not being a published *H. quentini* genome.

Whole-genomic DNA was extracted from *H. quentini* MP1 using a combination of QiaSymphony DSP DNA minikit (Qiagen, United Kingdom) and High Pure viral nucleic acid kit (Roche, United Kingdom). A library was prepared with Nextera DNA library preparation kit (Illumina), and sequenced with Illumina MiSeq (150-cycle Reagent Kit v3), which generated ~1.36 million 2 × 76 bp paired-end reads. Raw data quality was evaluated using FastQC (http://www.bioinformatics.babraham.ac.uk/projects/fastqc), and fastq-mcf (https://expressionanalysis.github.io/ea-utils/) was used to remove adaptors and low-quality sequences (<Q30). Clean data were assembled with Spades version 3.1.1 (7), and evaluated using QUAST (8). Short (<200 nucleotides) or low coverage contigs were removed, and a BLAST filter excluded a small number of contaminants with similarity to nonbacterial species. The final assembly consists of 2,161,515 bp in 97 contigs (averaging 98× coverage), with a *N*_50_ of 67,583. The largest contig is 216,255 bp in length, and the G+C content is 38.6%. Genome completeness was assessed using BUSCO (9). All 40 conserved bacterial genes are present in the assembly, one of which is duplicated.

It is hoped that by publishing this draft genome it will aid the identification and detection of *H. quentini* in clinical samples in the future. Further analysis of the genome, including comprehensive characterization, reference guided assembly, and comparison to both *H. haemolyticus* and *H. influenzae* published genomes is currently in progress to identify any distinct regions and genes.

### Accession number(s).

This whole-genome shotgun project has been deposited at GenBank under the accession no. MCII00000000. The version described in this paper is version MCII01000000.
